# Histopathology and levels of proteins in plasma associate with survival after colorectal cancer diagnosis

**DOI:** 10.1038/s41416-023-02374-z

**Published:** 2023-08-18

**Authors:** Magnus I. Magnusson, Bjarni A. Agnarsson, Jon G. Jonasson, Thordur Tryggvason, Famke Aeffner, Louise le Roux, Droplaug N. Magnusdottir, Helga S. Gunnarsdottir, Kristín K. Alexíusdóttir, Kristbjorg Gunnarsdottir, Emilia Söebech, Hjaltey Runarsdottir, Erna M. Jonsdottir, Bjarney S. Kristinsdottir, Sigurgeir Olafsson, Hildur Knutsdottir, Unnur Thorsteinsdottir, Magnus O. Ulfarsson, Daniel F. Gudbjartsson, Jona Saemundsdottir, Olafur T. Magnusson, Gudmundur L. Norddahl, J. E. Vivienne Watson, Thorunn Rafnar, Sigrun H. Lund, Kari Stefansson

**Affiliations:** 1 deCODE genetics/Amgen, Reykjavik, Iceland; 2 School of Engineering and Natural Sciences, University of Iceland, Reykjavik, Iceland; 3 Department of Pathology, Landspitali University Hospital, Reykjavik, Iceland; 4 Faculty of Medicine, University of Iceland, Reykjavik, Iceland; 5 Amgen Inc., South San Francisco, CA USA; 6 Department of Medicine, Landspitali University Hospital, Reykjavik, Iceland

**Keywords:** Colorectal cancer, Tumour biomarkers

## Abstract

**Background:**

The TNM system is used to assess prognosis after colorectal cancer (CRC) diagnosis. Other prognostic factors reported include histopathological assessments of the tumour, tumour mutations and proteins in the blood. As some of these factors are strongly correlated, it is important to evaluate the independent effects they may have on survival.

**Methods:**

Tumour samples from 2162 CRC patients were visually assessed for amount of tumour stroma, severity of lymphocytic infiltrate at the tumour margins and the presence of lymphoid follicles. Somatic mutations in the tumour were assessed for 2134 individuals. Pre-surgical levels of 4963 plasma proteins were measured in 128 individuals. The associations between these features and prognosis were inspected by a Cox Proportional Hazards Model (CPH).

**Results:**

Levels of stroma, lymphocytic infiltration and presence of lymphoid follicles all associate with prognosis, along with high tumour mutation burden, high microsatellite instability and *TP53* and *BRAF* mutations. The somatic mutations are correlated with the histopathology and none of the somatic mutations associate with survival in a multivariate analysis. Amount of stroma and lymphocytic infiltration associate with local invasion of tumours. Elevated levels of two plasma proteins, CA-125 and PPP1R1A, associate with a worse prognosis.

**Conclusions:**

Tumour stroma and lymphocytic infiltration variables are strongly associated with prognosis of CRC and capture the prognostic effects of tumour mutation status. CA-125 and PPP1R1A may be useful prognostic biomarkers in CRC.

## Background

Colorectal cancer (CRC) is the third most common cancer worldwide and the second most common cause of cancer death in the USA and in Europe [1, 2]. Similarly, in Iceland CRC accounted for 11.5% of all malignant tumours diagnosed in the year 2020 and was the second most common cause of cancer death [3].

The best established prognostic factors in colorectal cancer are the size and spread of tumour cells as reflected in the TNM (Tumour, Node, Metastasis) classification system and Dukes’ staging system [4, 5]. Both these systems take into account the invasion of the tumour into the surrounding tissue (T), spread to lymph nodes (N) and distant metastasis (M). TNM staging is a strong predictor for survival in stage I and IV disease, but is less accurate for stages II and III. Notably, patients with stages IIB or IIC have consistently been shown to have a worse prognosis than patients with stage IIIA [6–8]. Over the years, numerous factors not included in the TNM system have been associated with prognosis, notably, high tumour stroma levels have been reported as a poor prognostic factor, and high level of inflammatory cell infiltrate in tumour samples has been reported as a favourable prognostic factor [9–13]. Tertiary lymphoid structures have also been associated with a better prognosis in various cancers [14, 15]. Inflammatory parameters in tumour samples have received increased attention; specifically, a higher Immunoscore® assessment of CRC tumours has been shown to associate with a lower recurrence risk [16]. Anatomical locations of tumour have been shown to associate with prognosis, where generally, tumours located more proximally in the colon have been shown to have a worse prognosis [17, 18]. More recently, a more detailed anatomical division of colon subsegments showed a significant trend in worse prognosis from the left- to the right-sided colon [19].

Somatic mutations in the tumour genome have also been associated with prognosis. Higher tumour mutation burden (TMB, defined as number of somatic mutations per Mb) has been reported as a favourable prognostic factor [20], where a higher TMB is associated with a higher production of neoantigens, and therefore a stronger immune response [20]. Other studies have only associated high TMB status with survival in patients with *BRAF* mutations [21]. The effect of microsatellite instability (MSI) on prognosis has both been reported as non-significant and associated with better prognosis [19, 20, 22, 23]. The *APC*, *TP53* and *KRAS* genes are the most commonly mutated genes in CRC and have been found to acquire driver mutations in 72, 67 and 43% of tumours, respectively [24, 25]. Mutations in *KRAS* have been reported as an unfavourable prognostic factor [20, 26]. Conflicting results have been reported for mutations in *TP53* with some demonstrating a lack of association, whereas other reports describe an association with poorer prognosis [11, 20, 26]. Mutations in *APC*, which are found in over 70% of CRCs, have previously been described as a non-significant factor in prognosis [26]. *BRAF* mutations, found in approximately 10% of CRCs, have been shown to associate with a worse overall survival [27] and to only associate with survival in microsatellite stable tumours [28, 29].

Blood proteins have been investigated both as potential diagnostic and prognostic biomarkers in CRC. Whereas the search for a reliable diagnostic biomarkers has not been fruitful, blood levels of various proteins have been reported to associate with prognosis. As an example, serum levels of CA-125 (mucin-16), kallikrein-13, CEA (carcinoembryonic antigen), CA19-9 and γ-GT have been reported as prognostic factors in CRC [30–32]. However, no proteins are routinely used to assess prognosis in CRC patients.

Many of the aforementioned prognostic variables are strongly correlated; e.g. high tumour mutation load is strongly correlated with infiltration of immune cells into the tumour. It is therefore of importance to determine the correlations between molecular and histopathological variables in order to evaluate the independent effects these factors may have on survival. In this study, we looked at the correlation between histopathological features, tumour mutations and plasma protein levels along with their correlations with survival in CRC patients. As histopathological features, the amount of tumour stroma, absence or presence of lymphoid follicles (a form of tertiary lymphoid structures) and severity of lymphocytic infiltrate at the tumour margin were included in the analysis. TMB and MSI, along with mutations in *APC*, *TP53*, *KRAS* and *BRAF*, were included as examples of somatic mutations in the tumour samples. Measurements of 4963 plasma proteins were used to reflect the plasma protein features. We looked at the associations between the features in each group and survival, both individually and in a multivariate model adjusting for more conventional prognostic factors; i.e. TNM stage, age at diagnosis, tumour location (distal vs. proximal) and year of diagnosis.

## Materials and methods

### Data sources

**Fig. 1:**
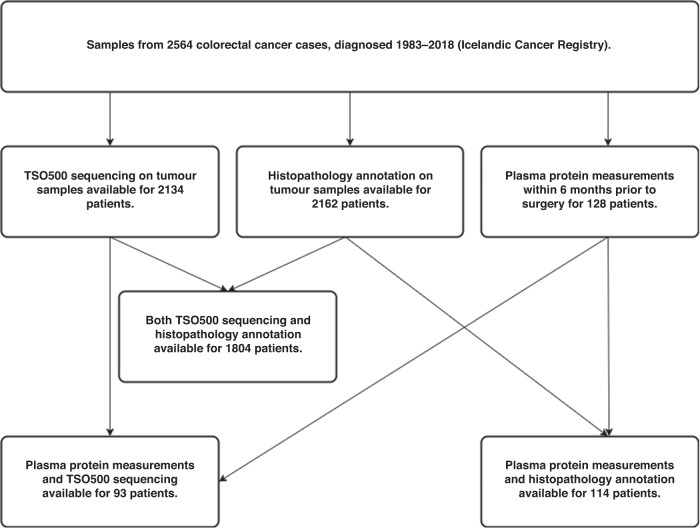
Data availability. Shown are the numer of CRC cases available for each type of analysis.

Information on all CRCs diagnosed in Iceland from 1983 to 2019 was extracted from the population-wide Icelandic Cancer Registry, including cancer stage as reflected by the TNM system, date of diagnosis, age at diagnosis, gender and whether the patient had previously been diagnosed with cancer. Information on the date of death and cause of death was collected from the Causes of Death Register, published by the Icelandic Directorate of Health (https://www.landlaeknir.is/english/). The follow-up time frame ended June 1 2021. The availability of measurements for the CRC cases is shown in Fig. 1.

Tumour samples were obtained from the biobank of Landspítali—The National University Hospital of Iceland (LSH). Information on Lynch syndrome in participants was extracted from the population-wide genotype database of deCODE genetics [33].

### Histopathological evaluation and scoring

**Fig. 2:**
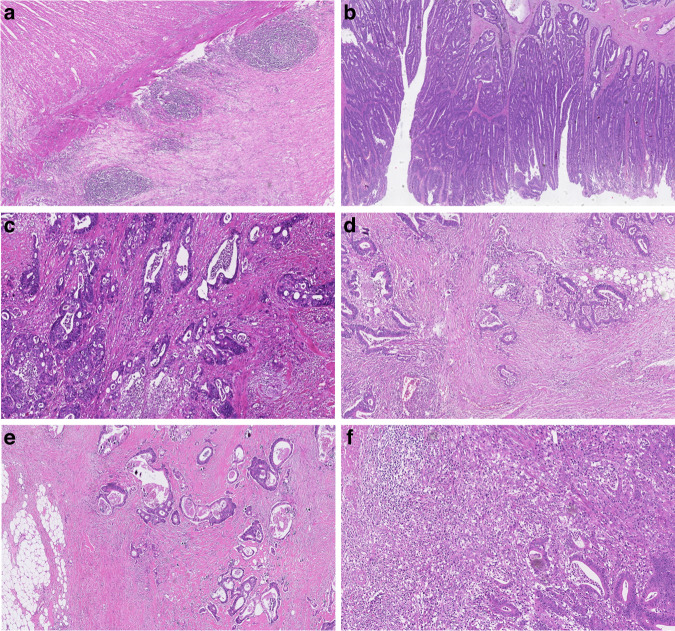
Representative examples of tumour samples. **a** lymphoid follicles in tumour sample; **b** tumour sample with a low (<10%) amount of stroma; **c** tumour sample with an intermediate (10–50%) amount of stroma; **d** tumour sample with a high (>50%) amount of stroma; **e** tumour sample with a low severity of lymphocytic infiltrate at the tumour margin; **f** tumour sample with a high severity of lymphocytic infiltrate at the tumour margin.

All biobank samples were reviewed by at least one out of three medical pathologists at LSH. All original haematoxylin and eosin (H&E)-stained sections of formalin-fixed paraffin-embedded (FFPE) resection samples were reviewed for tissue quality. The reviewing pathologist selected one slide per patient (if several were available) that was used for data collection. The following microscopic features were agreed upon at study start and captured per slide/patient: (1) the presence or absence of lymphoid follicles (absent/present) (Fig. 2a); (2) score of stroma amount in tumour (low = <10% stroma of total tumour area, intermediate = 10–50% and high = >50%) (Fig. 2b–d); and (3) score of severity of lymphocytic infiltrate at the tumour margin (low = no-to-mild lymphocytic infiltrate, high = moderate-to-marked lymphocytic infiltrate) (Fig. 2e, f). To facilitate consistent application of lymphocytic infiltrate scoring criteria, a reference guide with images was created at the onset of the project, which pathologists could revisit throughout the review period. No samples were reviewed by more than one pathologist. We compared the proportions of tumour samples classified into each group by each pathologist and found no significant difference in their proportions. The anatomical location of the tumours was classified as either proximal or distal, depending on their relative position with respect to the splenic flexure.

### Molecular analyses

The TruSight Oncology 500 panel (Illumina, Inc., San Diego, CA, USA) was used to evaluate tumour mutation burden (TMB; number of mutations/Mb), percentage microsatellite instability (%MSI) and presence of pathogenic mutations in the *KRAS*, *TP53*, *APC* and *BRAF* genes. DNA was isolated from a tissue core, 1 mm in diameter, taken through a tumour rich region of the FFPE tumour sample. DNA from the FFPE core was extracted on a chemagic Prepito instrument (PerkinElmer, Waltham, MA, USA, 2022-0020) using the Prepito FFPE Kit (PerkinElmer, CMG-2027). Briefly, the FFPE core samples were de-paraffinised in lysis buffer by heating to 90 °C for 5 min and cooled to room temperature prior to digestion by Proteinase K. Samples were incubated at 65 °C for at least 1 h or until tissue was fully lysed. Lysates were incubated at 90 °C for 45 min to reverse crosslinks prior to DNA isolation on the Prepito instrument.

Tumours were classified as either as having low TMB (TMB-L) (TMB ≤ 20 mutations/Mb) or high TMB (TMB-H) (TMB > 20 mutations/Mb). Furthermore, they were classified as either microsatellite low (MSI-L) (%MSI ≤ 15) or microsatellite high (MSI-H) (%MSI > 15). We note that in our data, all the MSI-H samples were also TMB-H. Mutations in *APC*, *TP53* and *KRAS* were classified as pathogenic based on the following criteria: *APC* and *TP53*; frameshifts, nonsense mutations, recurrent mutations reported in the COSMIC database [34]. *KRAS*; missense mutations at codons G12, G13, Q61, K117 and A146. For analysis of *BRAF*, we focused on the well-established oncogenic mutation in codon 600 (*BRAF* V600E). *BRAF* and *KRAS* mutations in cancers have been shown be mutually exclusive [35], and therefore, assessment of both with respect to survival is in order.

Plasma protein levels of 4,963 proteins were assessed with the SomaScan® assay (SomaLogic, Boulder, CO, USA). We restricted our analysis to plasma samples collected less than 6 months before initiation of treatment for CRC (128 patients). Given the widespread use of CA-125 in clinical applications, CA-125 levels in the same plasma samples were validated using Cobas (Elecsys CA 125 II, Roche diagnostics, Basel, Switzerland) and the association between these measurements and CRC-specific survival was assessed.

### Statistical methods

We analysed the association between the pathology variables and CRC-specific survival by using Cox regression. First, we fitted univariate models, where we inspected the association between CRC-specific survival and each histopathology variable separately, as well as for the other variables (sex, age at diagnosis, year of diagnosis, tumour location, TNM stage and Lynch syndrome status). The variables that were significantly associated with CRC-specific survival were then fitted together in a multivariate Cox regression. The relationship between the histopathologic variables and the somatic mutations in the tumour samples were assessed with a chi-squared test of independence. Association between CRC-specific survival and the somatic mutations was done similarly, first one variable at a time using Cox regression, and then in a multivariate model adjusting for stage, year of diagnosis, age at diagnosis, location and the pathologic features that were significantly associated with the somatic mutations. Plasma protein levels from the SomaScan® assay were adjusted for age and sex and normalised with inverse normal transformation [36]. Associations between plasma protein levels and CRC-specific survival were analysed using a univariate Cox regression, and a multivariate Cox regression, adjusting for stage, age at diagnosis and year of diagnosis. Hypothesis tests for all regression models were performed using likelihood ratio tests (LRT). All statistical analyses were performed using R (version 3.6.0). A *p* value of < 0.05 after adjustment for number of hypotheses tests was considered to indicate statistical significance. Adjustments for multiple comparisons were performed using Bonferroni´s method. The *p* value cutoff levels after Bonferroni adjustment for each analysis are included in their respective tables.

## Results

Patient characteristics of the CRC study group can be seen in Table 1.

**Table 1:** Descriptive statistics of the population samples.

	Histopathology annotation available (*N* = 2162)	TSO500 sequencing available (*N* = 2134)	Plasma protein data available (*N* = 128)
Age at diagnosis			
Mean (SD)	69.77 (12.26)	69.84 (12.19)	68.91 (11.83)
Year of diagnosis			
Mean (SD)	2003.25 (10.15)	2003.36 (10.00)	2003.23 (2.10)
Follow-up time			
Median (years)	4.58	4.50	9.58
Sex			
Male	1136 (52.5%)	1134 (53.1%)	73 (57.0%)
Female	1026 (47.5%)	1000 (46.9%)	55 (43.0%)
Prior cancer diagnosis			
No	1879 (86.9%)	1857 (87.0%)	126 (98.4%)
Yes	283 (13.1%)	277 (13.0%)	2 (1.6%)
Lynch syndrome			
No disease	2121 (98.1%)	2086 (97.8%)	126 (98.4%)
Disease	41 (1.9%)	48 (2.2%)	2 (1.6%)
Deceased			
Alive	673 (31.1%)	655 (30.7%)	35 (27.3%)
Deceased	1489 (68.9%)	1479 (69.3%)	93 (72.7%)
Deaths from CRC			
Alive/other	1442 (66.7%)	1424 (66.7%)	93 (72.7%)
Died from CRC	720 (33.3%)	710 (33.3%)	35 (27.3%)
Cancer stage (TNM)			
I	272 (12.6%)	247 (11.6%)	13 (10.2%)
II	801 (37.0%)	673 (31.5%)	52 (40.6%)
III	710 (32.8%)	614 (28.8%)	43 (33.6%)
IV	379 (17.5%)	310 (14.5%)	20 (15.6%)
Missing/uncertain	0 (0%)	290 (13.6%)	0 (0%)
Tumour location			
Proximal to splenic flexure	1014 (46.9%)	892 (41.8%)	51 (39.1%)
Distal to splenic flexure	1148 (53.1%)	1242 (58.2%)	64 (50.0%)
Missing	0 (0%)	0 (0%)	14 (10.9%)
Amount of stroma in the tumour			
<10%	768 (35.5%)	636 (29.8%)	56 (43.8%)
10–50%	841 (38.9%)	733 (34.4%)	30 (23.4%)
>50%	553 (25.6%)	466 (21.8%)	28 (21.8%)
Not available	0 (0%)	299 (14.0%)	14 (11.0%)
Lymphocytic infiltrate at the tumour margin			
Low–intermediate	1656 (78.4%)	1437 (67.3%)	87 (68.0%)
High	466 (21.6%)	398 (18.7%)	27 (21.0%)
Not available	0 (0%)	299 (14.0%)	14 (11.0%)
Lymphoid follicles			
Absent	1775 (82.1%)	1506 (70.6%)	94 (73.4%)
Present	387 (17.9%)	329 (15.4%)	20 (15.6%)
Not available	0 (0%)	299 (14.0%)	14 (11.0%)
TMB/Mb			
TMB > 20	294 (13.6%)	305 (14.3%)	14 (10.9%)
TMB ≤ 20	1541 (71.3%)	1829 (85.8%)	80 (62.5%)
Not available	327 (15.1%)	0	34 (26.6%)
%MSI			
%MSI > 15	269 (12.4%)	280 (13.1%)	11 (8.6%)
%MSI ≤ 15	1534 (71.0%)	1817 (85.1%)	82 (64.1%)
Not available	359 (16.6%)	37 (17.8%)	35 (27.3%)
APC mutation			
Present	1392 (64.4%)	1625 (76.1%)	64 (50.0%)
Absent	443 (20.5%)	509 (23.9%)	30 (23.5%)
Not available	327 (15.1%)	0	34 (26.5%)
KRAS mutation			
Present	769 (35.6%)	906 (42.5%)	38 (29.7%)
Absent	1066 (49.3%)	1228 (57.5%)	56 (43.8%)
Not available	327 (15.1%)	0	34 (26.5%)
TP53 mutation			
Present	1179 (54.5%)	1401 (65.7%)	69 (53.9%)
Absent	656 (30.4%)	733 (34.3%)	25 (19.6%)
Not available	327 (15.1%)	0	34 (26.5%)
BRAF mutation			
Present	327 (15.1%)	314 (14.7%)	17 (13.3%)
Absent	1744 (80.7%)	1820 (85.3%)	94 (73.4%)
Not available	91 (4.2%)	0 (0%)	17 (13.3%)

### Tumour stroma, lymphocytic infiltrate and the presence of lymphoid follicles independently associate with risk of death from CRC

**Fig. 3:**
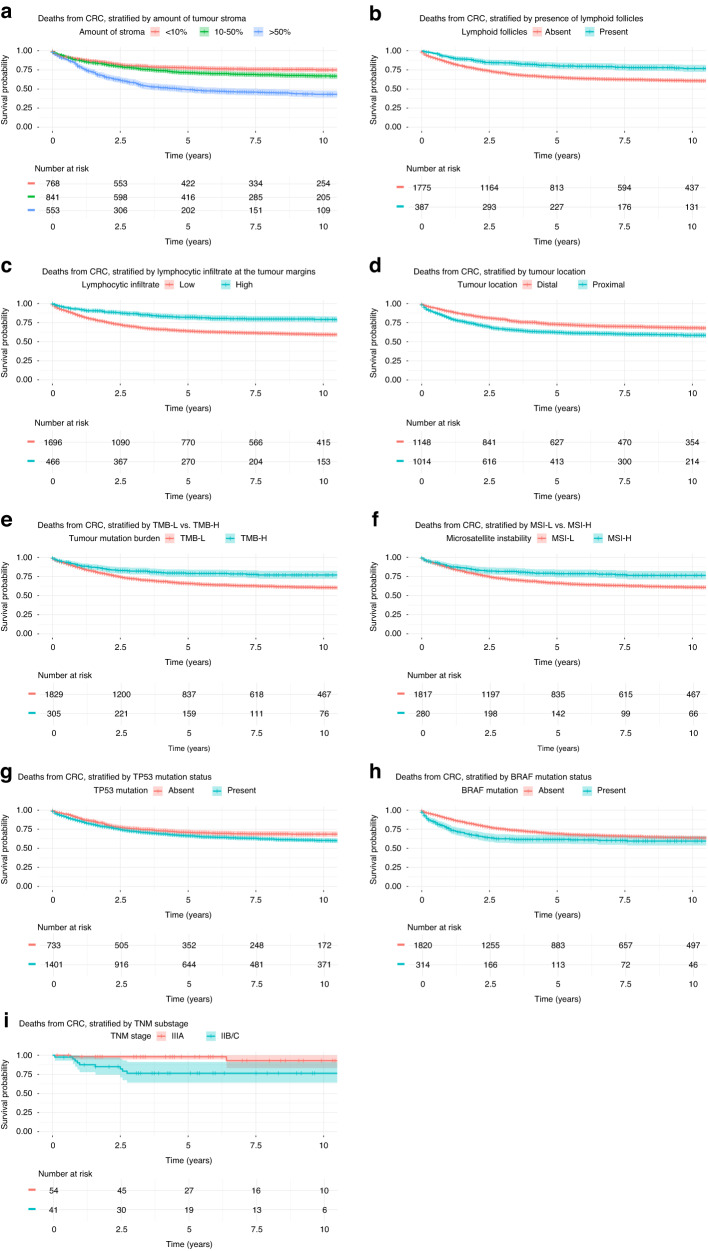
Kaplan–Meier curves of estimates of CRC-specific survival after CRC diagnosis. Stratification by **a** amount of tumour stroma; **b** presence of lymphoid follicles; **c** severity of lymphocytic infiltrate at the tumour margin; **d** proximal vs. distal tumour location; **e** TMB-L vs TMB-H; **f** MSI-L vs. MSI-H; **g** presence of pathogenic mutation in TP53; **h** presence of pathogenic mutation in BRAF; **i** stage IIB/C vs IIIA.

We first asked whether assessment of stroma and inflammatory features in the tumour are independent predictors of survival after CRC diagnosis. Univariate analysis of the histopathology features of the tumour (Table 2) shows that high level of stroma is strongly associated with increased risk of CRC death (HR = 2.71 for high amount, HR = 1.34 for intermediate amount of stroma, *p* < 2.2 × 10^–16^) (Fig. 3a). The presence of lymphoid follicles (HR = 0.51, *p* = 3.84 × 10^–10^) (Fig. 3b) and high lymphocytic infiltration at the tumour margin (HR = 0.44, *p* = 6.09 × 10^–13^) (Fig. 3c) are associated with lower risk of CRC death. We observed a significantly increased hazard ratio in tumours proximal to the splenic flexure relative to tumours distal to the splenic flexure (HR = 1.47, *p* = 2.7 × 10^–7^) (Fig. 3d). There was no association between survival and sex (HR = 1.00, *p* = 0.97) or prior cancer diagnosis (HR = 1.03, *p* = 0.79). Lynch syndrome (HR = 0.49. *p* = 0.023) was not significantly associated with survival after adjusting for the number of hypotheses tested. We fitted multivariate Cox regressions, including all covariates that were significant in the univariate Cox regressions, and estimated hazard ratios (HR) for the risk of death from CRC (Table 2). All histopathology features that were significant in the univariate analyses remained significant after adjustment for the other features, albeit with reduced effect sizes.

**Table 2:** Results from Cox proportional hazards models on deaths from colorectal cancers, fitted with the covariates in the table, in univariate Cox regression (left) and together in a multivariate model (right).

	Univariate analysis		Multivariate analysis	
	Hazard ratio (95% confidence interval)	*p* value	Hazard ratio (95% confidence interval)	*p* value
Intermediate amount of tumour stroma (10–50%)	1.34 (1.10–1.62)	<2.2 × 10^–16^***	1.18 (0.97–1.44)	1.18 × 10^–6^***
High amount of tumour stroma (>50%)	2.71 (2.25–3.27)		1.64 (1.35–2.00)	
High lymphocytic infiltration at the tumour margin	0.44 (0.35–0.55)	1.6 × 10^–15^***	0.74 (0.59–0.94)	0.0095*
Presence of lymphoid follicles in tumour	0.51 (0.40–0.64)	3.8 × 10^–10^***	0.68 (0.54–0.87)	0.001**
Tumour proximal to splenic flexure	1.47 (1.27–1.70)	2.7 × 10^–7^***	1.27 (1.09–1.47)	0.002**
Age at diagnosis	1.02 (1.01–1.03)	4.6 × 10^–8^***	1.03 (1.02–1.04)	<2.2 × 10^–16^***
TNM stage II	3.37 (1.98–5.74)	<2.2 × 10^–16^***	2.47 (1.45–4.22)	<2.2 × 10^–16^***
TNM stage III	7.79 (4.63–13.11)		6.16 (3.65–10.42)	
TNM stage IV	39.18 (23.26–65.97)		28.60 (16.84–48.54)	
Year of diagnosis	0.97 (0.96–0.97)	<2.2 × 10^–16^***	0.96 (0.96–0.97)	<2.2 × 10^–16^***
Female sex	1.00 (0.86–1.15)	0.97	Not included	
Lynch syndrome	0.49 (0.24–0.98)	0.02	Not included	
Prior cancer diagnosis	1.03 (0.82–1.29)	0.79	Not included	

*p* Values are results from likelihood ratio tests. For the univariate analysis, statistical significance is indicated with * for *p* values <5 × 10^–3^, ** for *p* values <5 × 10^–4^ and *** for *p* values <5 × 10^–5^ after adjustment for the number of hypothesis (10). For the multivariate analysis, statistical significance is indicated with * for *p* values <5 × 10^–2^, ** for *p* values <5 × 10^–3^ and *** for *p* values <5 × 10^–4^.

### Association between somatic mutations and survival is captured by the histopathology features

We assessed the relationship between somatic mutations (high/low TMB and high/low %MSI) and pathogenic mutations in *APC*, *TP53*, *KRAS* and *BRAF*) and the tumour pathology features (Supplementary Table 1). TMB-H tumours are associated with a lower amount of stroma (*p* = 5.42 × 10^–15^), higher amount of lymphocytic infiltration at the tumour margins (*p* = 9.47 × 10^–7^) and are more likely to have lymphoid follicles (*p* = 1.02 × 10^–14^). We observed that TMB-H tumours are more likely to located proximal to the splenic flexure (*p* < 2.2 × 10^–16^).

MSI-H tumours are likewise associated with a lower amount of tumour stroma (*p* = 2.40 × 10^–13^). Similarly to TMB-H tumours, they also associate with a higher inflammatory activity, i.e. they have a higher proportion of high amount of lymphocytic infiltration (*p* = 4.00 × 10^–7^) and a higher proportion of lymphoid follicles (*p* = 4.31 × 10^–15^). We similarly observed a higher proportion of MSI-H tumours in the proximal colon (*p* < 2.2 × 10^–16^).

Tumours with pathogenic *TP53* mutations are more likely to have higher amount of stroma (*p* = 6.8 × 10^–3^) and less likely to have lymphoid follicles (*p* = 9.0 × 10^–4^). *TP53* mutations are proportionally more prevalent in the distal colon (*p* = 4.79 × 10^–11^). We observed a similar location trend for *APC* mutations (*p* = 1.24 × 10^–8^).

*BRAF* mutations are associated with a lower amount of tumour stroma (*p* = 3.7 × 10^–4^). We observed a higher proportion of *BRAF* mutated tumours in the proximal colon (*p* < 2.2 × 10^–16^).

High tumour mutation load (TMB and/or MSI) as well as pathogenic mutations in *TP53*, *KRAS* and *BRAF* have been reported to associate with survival after CRC diagnosis. We tested this association in our patient population and specifically asked whether the mutation status was significantly associated with CRC-specific survival after adjustment for the histopathological features (Table 3). As previously reported, TMB-H (HR = 0.56, *p* = 1.64 × 10^–6^; Fig. 3e) and MSI-H (HR = 0.58, *p* = 1.62 × 10^–5^; Fig. 3f) statuses are associated with a better prognosis in a univariate Cox regression, whereas pathogenic mutations in *TP53* (HR = 1.31, *p* = 9.59 × 10^–4^; Fig. 3g) and *BRAF* (HR = 1.42, *p* = 9.50 × 10^–4^; Fig. 3h) are associated with a worse prognosis. However, none of those are significantly associated with survival when performing a multivariate Cox regression, adjusting for stage, year of diagnosis, age at diagnosis, location and the pathology variables.

**Table 3:** Associations between somatic mutations in tumour samples and survival.

	Univariate analysis		Multivariate analysis	
	Hazard ratio (95% confidence interval)	*p* value	Hazard ratio (95% confidence interval)	*p* value
High TMB (>20/Mb)	0.56 (0.43–0.72)	1.64 × 10^–6^***	0.75 (0.57–1.01)	0.051
High MSI (>15%)	0.58 (0.45–0.76)	1.62 × 10^–5^***	0.80 (0.59–1.07)	0.13
TP53 mutation	1.31 (1.11–1.54)	9.59 × 10^–4^*	1.12 (0.94–1.35)	0.20
KRAS mutation (driver)	1.17 (1.01–1.36)	0.037	Not included	
BRAF mutation	1.42 (1.16–1.74)	9.50 × 10^–4^*	1.16 (0.92–1.45)	0.21
APC mutation	0.84 (0.71–1.00)	0.053	Not included	

Univariate associations between somatic mutations in tumor samples and survival (left). Multivariate associations for each somatic mutation after adjusting for age at diagnosis, year of diagnosis, stage, location and tumour pathology (right). *p* Values are results from likelihood ratio tests. For the univariate analysis, statistical significance is indicated with * for *p* values <8.4 × 10^–3^, ** for *p* values <8.4 × 10^–4^ and *** for *p* values <8.4 × 10^–5^ after adjustment for the number of hypothesis (6). For the multivariate analysis, statistical significance is indicated with * for *p* values <5 × 10^–2^, ** for *p* values <5 × 10^–3^ and *** for *p* values <5 × 10^–4^.

Lastly, our data indicate that TMB-H are less likely to be diagnosed at stage IV than TMB-L tumours (OR = 0.31 for hypermutated tumours, *p* = 1.5 × 10^–6^). The same applies for MSI-H, which is a subset of TMB-H (OR = 0.31, *p* = 4.0 × 10^–6^).

### Multiple proteins associate with survival, most correlate with stage

To search for blood-borne proteins that associate with prognosis, we assessed the levels of 4963 proteins in plasma samples from 128 individuals that were collected within 6 months prior to surgical treatment of CRCs. There was no association between levels of any of the measured plasma protein levels and the histopathology variables (Supplementary Table 2). Furthermore, we observed no association between any of the protein levels and somatic mutations in the tumours (Supplementary Table 3). We observed an elevated level of one protein, Transferrin receptor protein 1 (TfR1), in proximally located tumours versus in distally located tumours (proximal mean = 1.66, distal mean = 0.21, *p* = 4.21 × 10^–8^).

We identified 14 proteins that associate with survival when performing a univariate Cox regression (Supplementary Table 4). After adjusting for stage, year of diagnosis, and age at diagnosis, we observed two proteins that associate with survival. These are CA-125 (HR = 2.19, *p* = 4.34 × 10^–6^) and PPP1R1A (HR = 2.53, *p* = 5.11 × 10^–6^). Given that CA-125 is commonly measured in the clinical setting for cancer monitoring, we repeated the CA-125 measurements using Cobas (Elecsys CA 125 II, Roche diagnostics) –the method used by LSH. Here, we did not adjust the CA-125 levels for age, sex nor did any transformation of the measurements, but instead used the raw levels of CA-125 (U/mL). Again, CA-125 was significantly associated with survival (HR = 1.028, *p* = 2.83 × 10^–6^) in a univariate analysis, and after adjusting for TNM stage, age, and year of diagnosis (HR = 1.22, *p* = 1.1 × 10^–4^).

### Histopathologic features, CA-125 and PPP1R1A levels are strongly associated with survival in patients with stage II and III disease

TNM staging is the strongest factor used for determining post-operative treatment of CRC cases. While TNM staging accurately predicts survival for stage I and IV patients, improvements in the evaluation of prognosis for cases with stage II and III disease are needed. We performed a subgroup analysis for this group where we inspected the association between each of the histopathology features on CRC-specific survival, while adjusting for location, age and year of diagnosis. Tumour stroma amount is associated with survival in both the stage II and stage III groups (Supplementary Table 5). The presence of lymphoid follicles is significantly associated with survival in the stage II group and high severity of inflammation at the tumour margin is significantly associated with survival in the stage III group (Supplementary Table 5).

Previous studies have consistently shown that locally invasive tumours without lymph node involvement, stage IIB (T4a, N0) and stage IIC (T4b, N0), have worse prognosis than less locally advanced tumours with lymph node involvement (stage IIIA; T1/2, N1 or T1, N2a) [6–8]. Since we observed a strong association between the histopathologic features and prognosis, we asked whether those features associate with the local invasion of the tumours. Focusing only on the cases that have less invasive tumours with lymph node involvement (Stage III A) or more invasive tumours without lymph node involvement (Stage IIB/C) (95 individuals), i.e. the groups where the “Survival paradox” has been described, we also observe a worse prognosis for the stage IIB/C group than stage IIIA (HR = 6.41, *p* = 0.0057; Fig. 3i). Notably, the more locally invasive stage IIB/C tumours are more likely to have high amount of stroma (OR = 4.62, *p* = 0.0047) and less likely to have high amount of lymphocytic infiltrate at their margins (OR = 0.20, *p* = 0.0070).

For CRC cases with stage II or III disease and plasma protein measurement (*n* = 95), we found both CA-125 and PPP1R1A to associate with survival after adjusting for stage (II vs III), year of diagnosis and age at diagnosis (HR = 2.26, *p* = 0.0013 for CA-125, HR = 1.99, *p* = 0.0035 for PPP1R1A).

## Discussion

In the clinical setting, the TNM and Dukes’ staging systems are the most commonly used tools to assess prognosis in colorectal cancers. They do, however, only take into account a limited number of factors known to affect survival. Furthermore, the widely used TNM system has been shown to give unreliable guidance on prognostication, where numerous studies have shown worse prognosis for stage IIB/C patients than for those with stage IIIA [6–8]. While histopathological features, somatic mutations in tumours and plasma protein levels have been identified as potential measures of prognosis, the association between them and their combined association with outcome has not previously been assessed.

In this study, we have used patient outcomes from the population-wide cancer registry in Iceland and both pathological assessment and genome sequencing of tumour samples. The sample size in our study allowed for the combined assessment of multiple features on prognosis and the correlation between these features.

We saw a strong association between worse prognosis and higher stroma amount in tumours, and an association between presence of lymphoid follicles and higher amount of lymphocytic infiltrate at the tumour margins and a better prognosis. Tumour stroma, that is mainly composed of connective tissue cells and extracellular matrix, provides support for the maintenance and growth of tumour cells, and high tumour stroma has previously been identified as an independent indicator of worse prognosis [9, 10]. The role of tumour stroma in drug resistance in solid tumours has also been described [37]. In the past years, the significance of how immune response in tumour samples affects prognosis has been firmly established [11–13]. The role of tertiary lymphoid structures or aggregates of lymphocytes, such as lymphoid follicles, in antitumour activity is a rich field of study. The association between tertiary lymphoid structures and a better prognosis has sparked research into the possible roles they may have in antitumour immunity [14, 15]. The three histopathological assessments used here all have a significant independent correlation with CRC-specific survival, suggesting that each of these features may play a distinct role in the pathogenesis of CRC.

In order to aid prognostication, investigators have sought to test whether somatic mutations in tumours associate with course of disease and survival. In our study, we observed associations between both TMB-H and MSI-H and a better prognosis. Whereas previous studies have shown an association between tumour mutation burden and survival both independently [20] and in relation to other somatic mutations, e.g. *BRAF* mutations [21], our data indicates that the TMB status and tumour histopathology features are highly correlated, and that TMB status does not significantly associate with survival when adjusted for them. [20]Similarly, while the prognostic association of microsatellite instability has been widely reported in the literature, we also note that microsatellite instability is strongly correlated with tumour histopathology, and similar to TMB, MSI is not significantly associated with prognosis when the pathological assessments are accounted for. For the oncogenic mutations, *TP53* mutations were associated with a worse prognosis, as previously described [38, 39], in a univariate analysis. *BRAF* mutations were similarly associated with a worse prognosis in a univariate analysis, like reported elsewhere [27]. However, we also observed a significant association between these oncogenic mutations and the pathological assessments, and neither were significantly associated with survival after correcting for the pathological assessments of the tumours together with disease stage, location, year of diagnosis and age at diagnosis.

We noticed that TMB-H and MSI-H tumours were less likely to be diagnosed at stage IV, as previously reported in [38]. This is possibly due to the strong association between the level of somatic mutations in the tumours and inflammation of the surrounding tissue which may in turn lead to earlier symptoms.

Focusing only on stage II and III patients, we also observe the “survival paradox”, i.e. stage IIB/C patients seem to have a worse prognosis than stage IIIA patients [6–8]. We also observe that the less locally invasive tumours in the stage IIIA group are more likely to have higher lymphocytic infiltration at their margins and less amount of stroma. Since both of these factors associate with survival, this might provide insight into the underlying cause of the “survival paradox”, i.e. a microenvironment favourable to tumour proliferation might have a more impact on disease progression than a spread to a few local lymph nodes

We observe a positive correlation between the levels of two plasma proteins, CA-125 and PPP1R1A, and worse prognosis, even after adjusting for the TNM stage of the disease. CA-125 is most commonly known for its use in monitoring the progression of ovarian cancer. However, CA-125 has recently been described as a prognostic marker for CRC survival, where it was reported that levels of CA-125 outperform levels of CEA and CA-19.9 when assessing prognosis [30]. Furthermore, CA-125 has been reported as a possible biomarker for peritoneal dissemination of CRC [40]. Our results provide additional evidence for the use of this marker as a prognostic marker. We also identified an association between levels of PPP1R1A and worse prognosis. PPP1R1A is an inhibitor of protein-phosphatase-1 and has previously been linked to carcinogenesis [41]. The effect of PPP1R1A on the progression and metastasis of Ewing’s sarcoma has previously been described [42]. To the best of our knowledge, the protein has not previously been associated with prognosis in CRC and further validation is needed. Our finding that these two proteins are associated with survival in the stage II and III group indicates that measurements of their plasma levels could further add to prognostic assessments in this group.

None of the plasma proteins measured associate with either the histopathology variables or the somatic mutations in the tumour samples. The prognostic value of CA-125 and PPPR1A1 levels could therefore be reflecting response to the tumour rather than the properties of the tumour and/or the immediate tumour microenvironment.

A single protein, Transferrin receptor protein 1 (TfR1), was elevated in patents with proximally sided tumours versus patients with distally sided tumours. Serum levels of Transferrin receptors have been shown to be elevated in subjects with iron deficiency anaemia [43]. The elevated levels of TfR1 in patients with proximally located tumours in our study group could therefore reflect a longer duration of, or a heavier, bleeding from the tumour site.

To conclude, to the best of our knowledge we report here the analysis of the largest set of data on colorectal tumours with tumour histopathology, tumour genetics and patient outcome (*n* > 2000 cases).

We acknowledge that having a larger sample set for the plasma protein analyses would have strengthened the results. While CA-125 has previously been associated with prognosis in colorectal cancers, the association between PPPR1A1 might be by chance. Another limitation is that all our MSI-H samples were also TMB-H.

Building upon findings in the literature, we provide evidence that assessment of stroma amount and lymphocytic infiltration should be taken into account when assessing prognosis of CRC. The prognostic value of plasma levels of CA-125 that we observe in our data provides information not captured by the histopathology variables. Given the widespread use of CA-125 testing as a monitoring marker for ovarian cancer, its potential for use to assess prognosis of CRC should be further explored.

### Supplementary information

Supplementary tables

REMARK checklist

## Data Availability

The data that supports the findings of this study are available in the Supplementary Material of this article.
